# Spatio-temporal spread of artemisinin resistance in Southeast Asia

**DOI:** 10.1371/journal.pcbi.1012017

**Published:** 2024-04-16

**Authors:** Jennifer A. Flegg, Sevvandi Kandanaarachchi, Philippe J. Guerin, Arjen M. Dondorp, Francois H. Nosten, Sabina Dahlström Otienoburu, Nick Golding

**Affiliations:** 1 School of Mathematics and Statistics, University of Melbourne, Melbourne, Australia; 2 WorldWide Antimalarial Resistance Network (WWARN), Oxford, United Kingdom; 3 CSIRO’s Data 61, Melbourne, Australia; 4 Infectious Diseases Data Observatory (IDDO), Oxford, United Kingdom; 5 Centre for Tropical Medicine and Global Health, Nuffield Department of Medicine, University of Oxford, Oxford, United Kingdom; 6 Mahidol Oxford Tropical Medicine Research Unit, Faculty of Tropical Medicine, Mahidol University, Bangkok, Thailand; 7 Shoklo Malaria Research Unit (SMRU), Mahidol-Oxford Tropical Medicine Research Unit (MORU), Faculty of Tropical Medicine, Mahidol University, Mae Sot, Thailand; 8 College of Science, Technology, Engineering and Mathematics, Johnson C. Smith University, Charlotte, North Carolina, United States of America; 9 Telethon Kids Institute and Curtin University, Perth, Australia; University of Zurich, SWITZERLAND

## Abstract

Current malaria elimination targets must withstand a colossal challenge–resistance to the current gold standard antimalarial drug, namely artemisinin derivatives. If artemisinin resistance significantly expands to Africa or India, cases and malaria-related deaths are set to increase substantially. Spatial information on the changing levels of artemisinin resistance in Southeast Asia is therefore critical for health organisations to prioritise malaria control measures, but available data on artemisinin resistance are sparse. We use a comprehensive database from the WorldWide Antimalarial Resistance Network on the prevalence of non-synonymous mutations in the Kelch 13 (K13) gene, which are known to be associated with artemisinin resistance, and a Bayesian geostatistical model to produce spatio-temporal predictions of artemisinin resistance. Our maps of estimated prevalence show an expansion of the K13 mutation across the Greater Mekong Subregion from 2000 to 2022. Moreover, the period between 2010 and 2015 demonstrated the most spatial change across the region. Our model and maps provide important insights into the spatial and temporal trends of artemisinin resistance in a way that is not possible using data alone, thereby enabling improved spatial decision support systems on an unprecedented fine-scale spatial resolution. By predicting for the first time spatio-temporal patterns and extents of artemisinin resistance at the subcontinent level, this study provides critical information for supporting malaria elimination goals in Southeast Asia.

## Author summary

Resistance to artemisinin derivatives has been confirmed in the Greater Mekong Subregion, with worrying signs of spread in India and more recently emergence in Rwanda and Uganda. This situation is dire given the way that the emergence and spread of resistance to other antimalarial drugs, chloroquine and later sulphadoxine–pyrimethamine, resulted in dramatic increases in malaria-related morbidity and mortality across sub-Saharan Africa in the 1990s. To eliminate malaria, up-to-date maps of artemisinin resistance are urgently needed; predictive models of the spread of drug resistance can make far-reaching, significant, changes in our approach to malaria elimination by informing appropriate changes to drug policy. In this study, we have provided the first data-driven, predictive maps of the changing landscape of resistance to artemisinin derivatives in the Greater Mekong Subregion. These maps provide estimates where no data are available and can be used by health agencies to guide the prioritisation of surveillance for resistance, and policies to improve treatment and prevent the further spread of resistance.

## Introduction

Antimalarial drugs are essential tools for the control and elimination of malaria. Resistance to all currently available antimalarials, including the pivotal artemisinin derivatives, have been confirmed. This situation is dire considering how the multi-foci emergence and spread of resistance to other antimalarial drugs–chloroquine and, later, sulphadoxine–pyrimethamine (SP)–have resulted in dramatic increases in malaria-related morbidity and mortality [1].

Studies conducted in 2006–2007 first reported that *P*. *falciparum* in north-west Cambodia had reduced in vivo susceptibility to artemisinins, which manifested as delayed clearance of parasites from the blood of patients treated with ACTs [2]. Delayed parasite clearance in *P*. *falciparum* infections following artemisinin-based therapies is the clinical hallmark of resistance [3]. *P*. *falciparum* infections with significantly slowed parasite clearance under artemisinin-based therapies have now been detected in neighbouring countries in the Greater Mekong Subregion; namely in Vietnam, Thailand and Myanmar [4,5].

Spatial information on changes to antimalarial resistance is critical for health organisations to prioritise control measures, but available data on artemisinin resistance are sparse. No such fine-scale predictive maps exist for artemisinin resistance within Southeast Asia. However, to generate predictive maps using appropriate model-based geostatistics, large numbers of unique spatio-temporal locations are needed [6]. This is impossible to achieve with clinical measures of resistance; the data are simply not available owing to the expensive and time-consuming nature of clinical studies [4,7]. A way forward is to use molecular markers associated with resistance.

Genetic mutations known to be associated with drug resistance can be used to monitor spatio-temporal trends in antimalarial drug resistance as a proxy of clinical efficacy. Genetic studies are easier to conduct and are a fraction of the cost of clinical studies, thereby allowing larger numbers of samples to be collected across more spatio-temporal locations [7]. Data from genetic studies are readily amenable to model-based geostatistics. In a previous study, Flegg *et al*. developed a predictive model for the geographical and temporal trends across Africa of the prevalence of mutations in the *dhps* gene of the parasite that are known to be associated with SP resistance [8]. A continuous predictive surface in space and time was inferred for *dhps* markers using Bayesian model–based geostatistics over the spatial domain of sub-Saharan Africa from 1990 to 2010. This has since been extended with more data to make more recent predictions up until 2020 [9]. These models were built in a Bayesian framework so that predictions can be informed not only by the observed data but also by prior knowledge. The maps have proved useful to policymakers, and the predictive maps of SP resistance were combined with clinical data on low birthweight [10]. The analysis showed that SP should be considered compromised, and countries should switch to alternative strategies if the *dhps*-A581G marker is prevalent at more than 30%.

In 2014, mutations in the propeller region of the K13 protein that are associated with slow parasite clearance response to artemisinins were identified and almost 200 different candidate mutant genotypes were established [11]–a list that has been subsequently revised [12]. Since the identification of K13 markers, many studies have been conducted (and historical blood samples reanalysed) so that now a significant amount of data exists on the prevalence of these markers.

In this paper, data-driven, predictive spatio-temporal maps are presented for the first time of the changing landscape of resistance to artemisinin derivatives across the Greater Mekong Subregion. We develop a geostatistical model of the presence of molecular markers associated with artemisinin resistance, in the Kelch 13 (K13) gene, calibrated to the most comprehensive dataset available from the WorldWide Antimalarial Resistance Network (WWARN). The spatio-temporal maps of K13 marker prevalence presented in this paper will be able to support monitoring of drug resistance in Southeast Asia, appropriate targeting of preventive strategies and malaria elimination efforts.

## Methods

We use a Bayesian model–based geostatistics approach to create predictive maps of K13 marker prevalence. Predictions at a spatial location are influenced by (1) the distance in space and time to all available data and (2) a set of spatio-temporal covariates in the regression sub-model for the mean of the stochastic process. A brief overview is given below with details on the covariate data, geostatistical model, clustering and validation provided in the Supplementary Methods in S1 Text.

### Kelch 13 data and model

We use data publicly available through WWARN’s comprehensive artemisinin molecular surveyor [13], which collates the prevalence of molecular markers in the propeller region of the K13 gene of the malaria parasite. All non-synonymous mutations at any K13 locus > 440 (i.e., within the propeller region) are captured along with a date of sample collection and geospatial information. In some studies, a non-synonymous mutation in locus 252, outside the propeller domain, was captured. Data were included from diverse types of studies, for example, cross-sectional studies and therapeutic efficacy trials; however, any studies whose samples were collected after treatment with an antimalarial were not included. Note that the spatial location refers to the sample collection site, which in some instances could differ from the location of infection. For example, artemisinin-resistant *P*. *falciparum* infections may have been detected in Thailand, resulting from movement across borders from Cambodia and Myanmar [14]. Only studies where an individual site location could be determined (e.g. a single village) were included in the study; for any aggregated data (e.g. across multiple villages), the data contributors were contacted, and individual site locations were sourced. We downloaded data from WWARN’s Artemisinin Molecular Surveyor on 7 June 2023.

**Fig 1:**
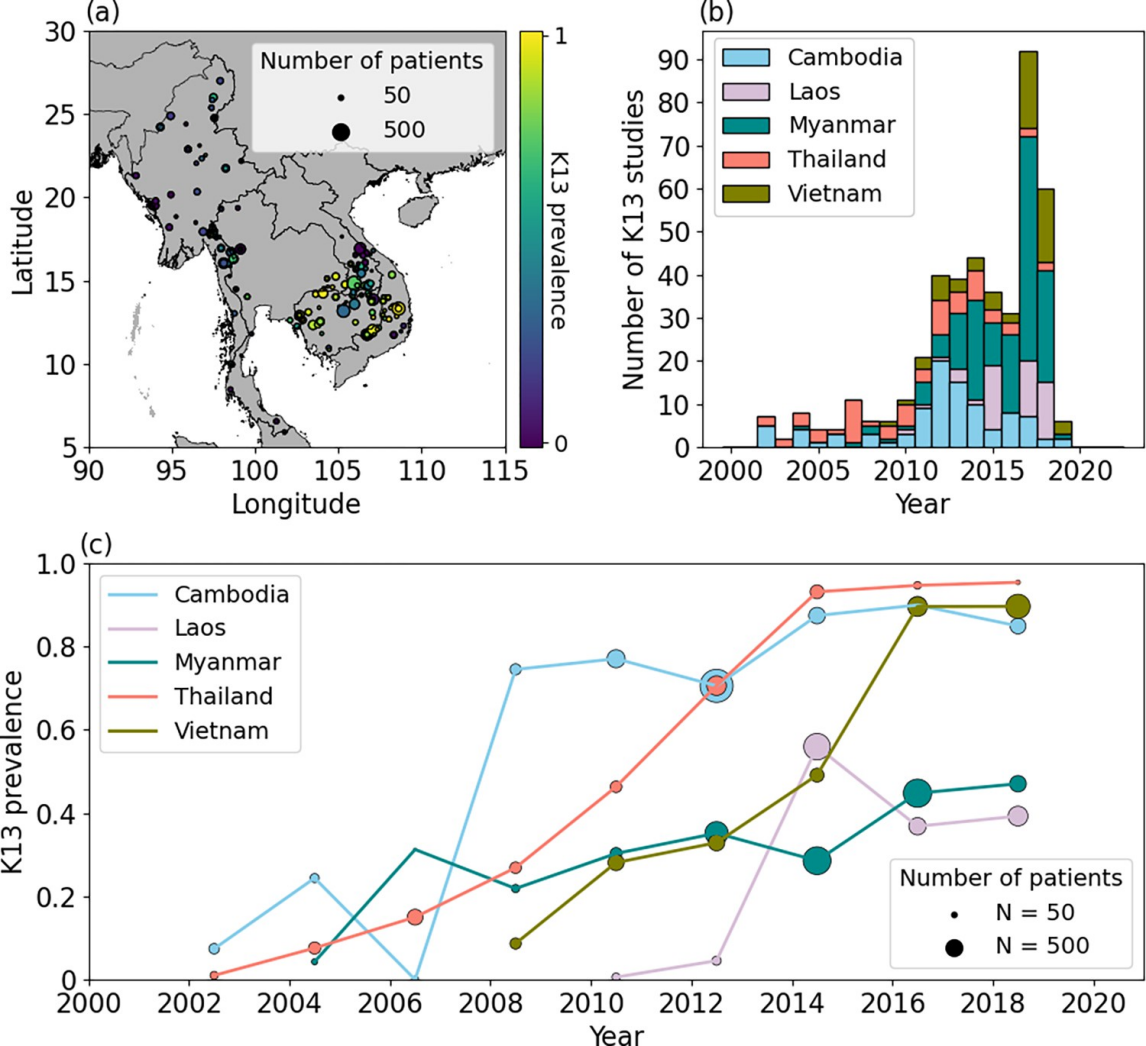
(a) Map showing the spatial location of the studies included in the modelling of K13 prevalence. The size of the marker is proportional to the number of patients in the study and the colour of the marker denotes the observed K13 marker prevalence. National shapefiles were obtained from the Malaria Atlas Project (MAP; https://malariaatlas.org/) under their open access policy (https://malariaatlas.org/open-access-policy/) and no changes were made. (b) Number of studies each year since 2000, broken down by country. (c) Temporal trends in country level K13 prevalence data since 2000, where the number of patients is denoted by the size of the circles.

In this work, we model the overall prevalence of any K13 mutation that has been associated with delayed parasite clearance [12]. That is, a sample is classified as ‘positive’ if it has a mutation at any K13 locus listed in Table 1 and classified as ‘negative’ otherwise. For a given study, the prevalence of K13 mutations is then defined as the proportion of positive K13 samples out of those tested. We include only studies with a sample size of at least 10. Fig 1A shows the spatial locations of 431 studies included in the analysis; the studies were conducted in Cambodia, Laos, Myanmar, Thailand and Vietnam (Fig 1B). In each of these countries, the trend of K13 marker prevalence has risen since the early 2000s (Fig 1C). S1 Video shows the time course of K13 data collection over the period of 2000 to 2022, for which the data visualised in each year shows studies conducted before or during the year associated with the map.

**Table 1:** *K13* mutant alleles that have been strongly associated with slow parasite clearance from [12], that are considered here for spatio-temporal mapping.

N458Y	Y493H	R539T	I543T	R561H	C580Y	E252Q	P441L
F446I	G449A	M476I	A481V	R515K	P527H	N537I	G538V
P553L	V568G	P574L	P667T	A675V	R539R/T	C580C/Y	

## Results

We use the K13 molecular marker data detailed in the Methods to calibrate the geostatistical model (see S1 Text for details). The four covariates used in the model are malaria parasite rate (posterior median regression coefficient of -0.78), human population density (-0.015), malaria temperature suitability (0.071), and travel time to nearest city as a measure of accessibility (-0.024).

The calibrated geostatistical model allows quantification of the predicted distribution of K13 prevalence for any location of interest in any year in the Greater Mekong Subregion. It is important to note that these maps do not represent resistance in malaria cases being transmitted at each location, but rather the malaria cases presenting at these locations (in that the location where malaria is transmitted by mosquito bite may be quite different to the location where the malaria case presents due to travel of the infected person).

### Model validation

Overall, there is good agreement between the observed (in hold-out data) and predicted prevalence (correlation coefficient of 0.82). There is a small amount of bias (mean error of −0.0035), and the accuracy is reasonable (mean absolute error of 0.13). Fig G in S1 Text shows good agreement between the observed and predicted prevalence, and reliability of the credible intervals is strong, especially for narrow intervals. When we perform a spatially explicit cross validation with the data spatially clustered into 50 clusters, there is still reasonable agreement between observed and predicted prevalence (correlation coefficient of 0.56, mean error of -0.058 and mean absolute error of 0.22).

### Kelch 13 marker prevalence over time

**Fig 2:**
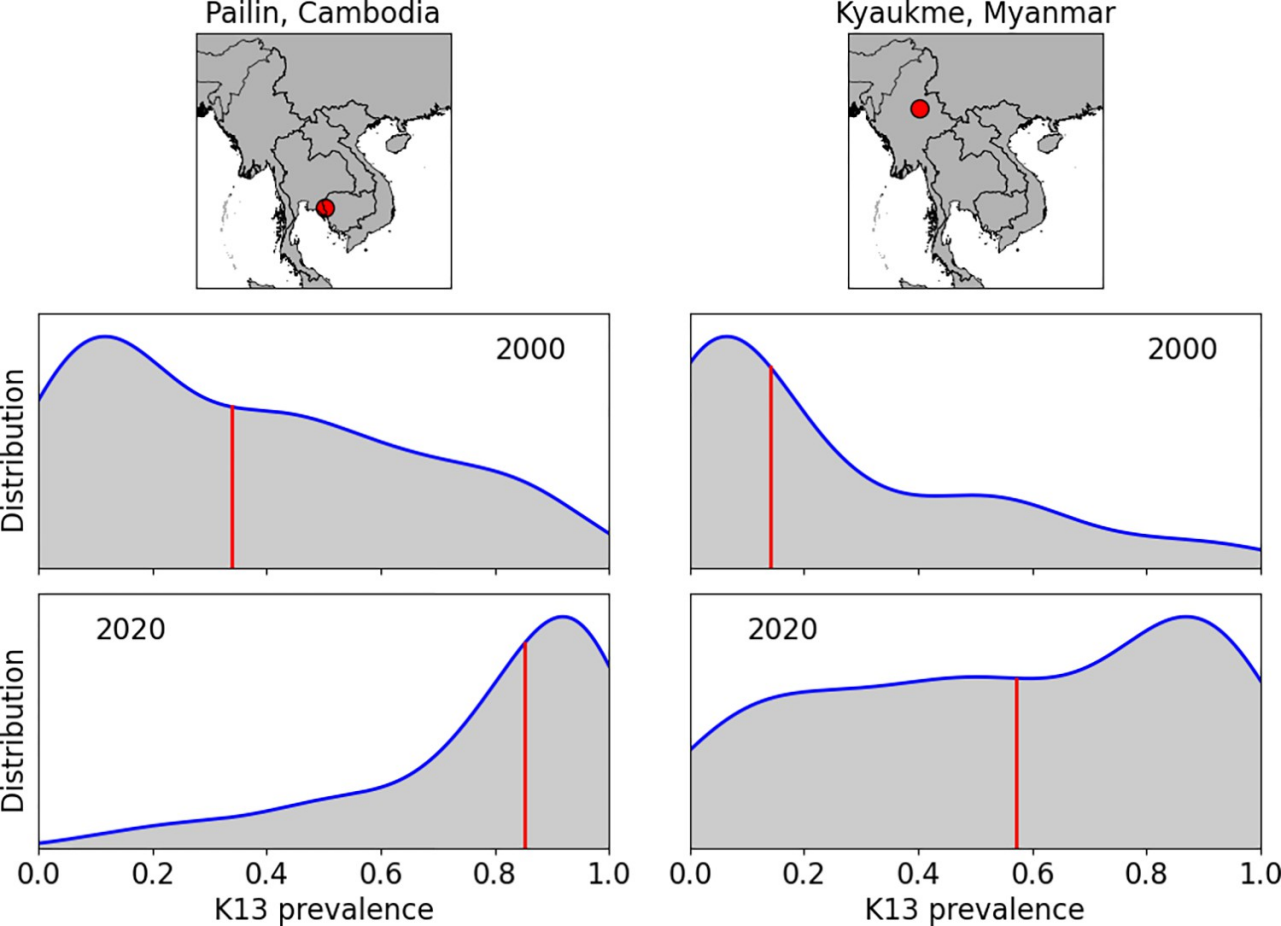
Example posterior predictive distributions for K13 prevalence at two locations, in Pailin, Cambodia (left hand side panels) and Kyaukme, Myanmar (right hand side panels). The rows relate to the predictive distribution in 2000 and 2020, respectively. The vertical red lines represent the median prevalence. National shapefiles were obtained from the Malaria Atlas Project (MAP; https://malariaatlas.org/) under their open access policy (https://malariaatlas.org/open-access-policy/) and no changes were made.

Fig 2 compares the predicted distributions at two locations: Pailin, Cambodia (left-hand side), and Kyaukme, Myanmar (right-hand side), in 2000 (second row) and 2020 (third row). The complete posterior predictive distribution is presented, and the median is shown as a solid red vertical line. In 2000, the predicted level of K13 prevalence is already high in Pailin compared with Kyaukme. By 2020, both locations have increased predicted levels of resistance compared with 2000.

**Fig 3:**
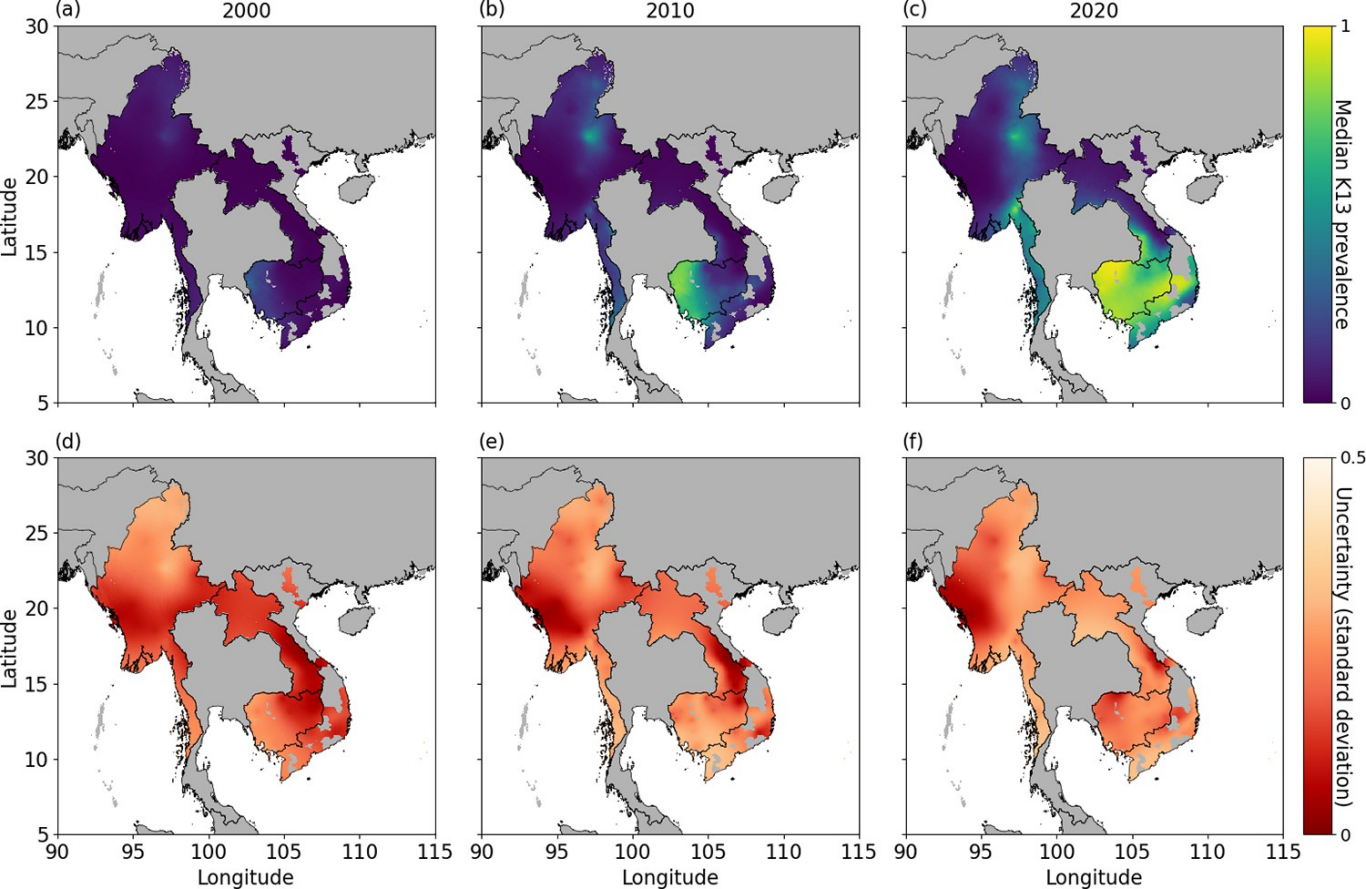
Posterior predictive median prevalence of K13 marker in the Greater Mekong Subregion in 2000 (a), 2010 (b) and 2020 (c), in regions where the median MAP estimates of parasite rate among those aged 2–10 years is predicted to be non-zero are visualised (see Fig C in S1 Text for results for the entire Greater Mekong subregion). Associated standard deviations for posterior predictions in 2000 (d), 2010 (e) and 2020 (f). National shapefiles were obtained from the Malaria Atlas Project (MAP; https://malariaatlas.org/) under their open access policy (https://malariaatlas.org/open-access-policy/) and no changes were made.

The posterior predictive distribution of K13 prevalence is obtained on a 5 x 5 km grid in the Greater Mekong Subregion from 2000 to 2022. The median and uncertainty (standard deviation) of the distribution are shown in Fig 3 for the Greater Mekong Subregion in 2000, 2010 and 2020, in regions where the median estimate of parasite rate among those aged 2–10 years is predicted to be non-zero (see Fig C in S1 Text for results over the entire Greater Mekong Subregion). We note that we could exclude regions based on other measures of malaria transmission that are more likely to reflect disease burden in the region accurately; however, parasite rate is the only metric publicly available at an appropriate spatial and temporal scale.

In locations where resistance is predicted to be established in 2000 already (e.g., Thai–Cambodia border), resistance levels are predicted to have increased (spread out or emerged) geographically in neighbouring regions (compare Fig 3A–3C for 2000, 2010 and 2020, respectively). The associated uncertainties show a general increase in uncertainty from 2000 to 2020 (compare Fig 3D–3F for 2000, 2010 and 2020, respectively). S2 and S3 Videos show the median and uncertainty, respectively, of the posterior predictive distribution of K13 prevalence over 2000–2022.

### Predicted extent of Kelch 13 mutations

**Fig 4:**
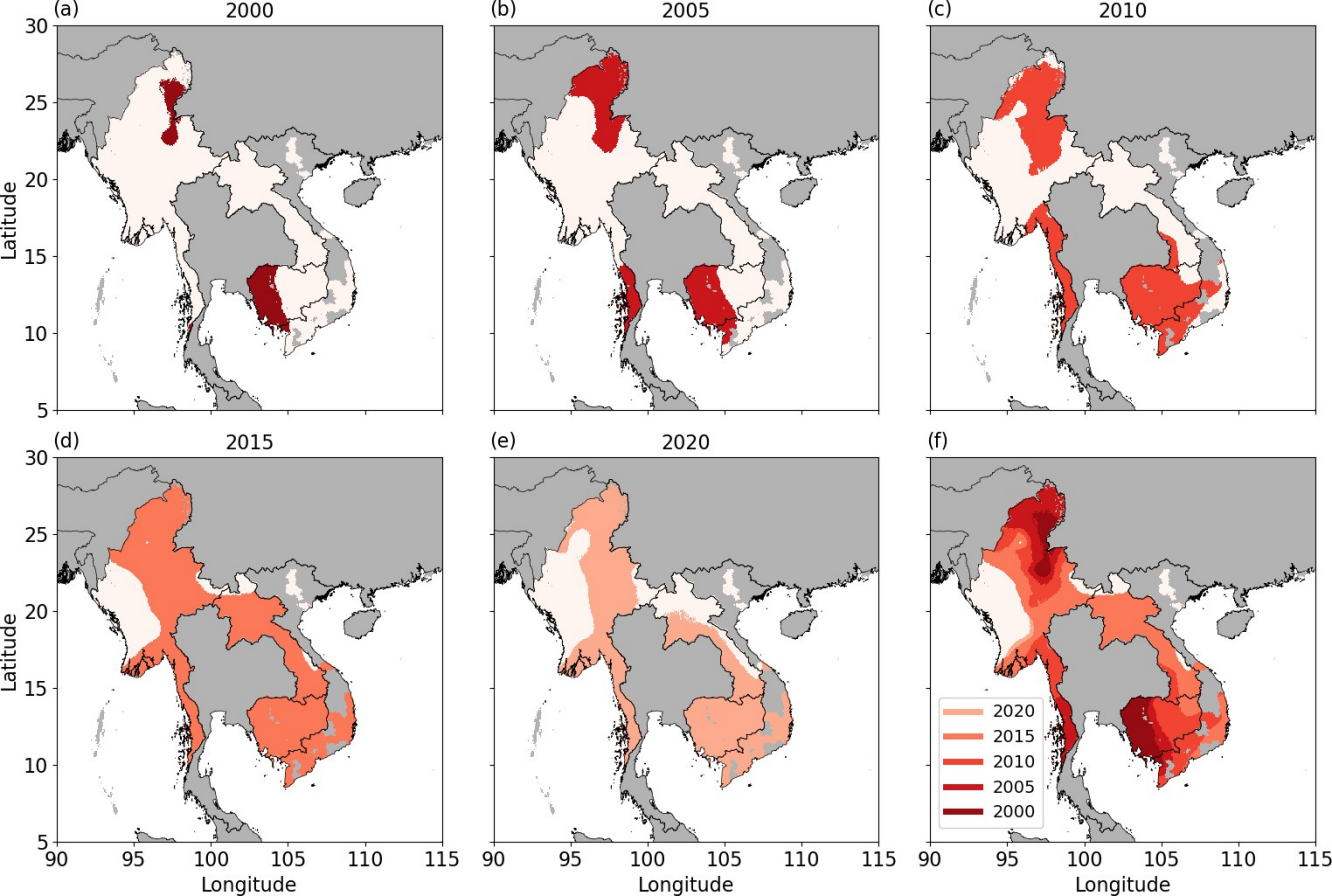
The predicted area with K13 marker prevalence exceeding 10% (shaded region), based on median predictions, in 2000 (a), 2005 (b), 2010 (c), 2015 (d) and 2020 (e), in regions where the median MAP estimates of parasite rate among those aged 2–10 years is predicted to be non-zero are visualised (see Fig D in S1 Text for results for the entire Greater Mekong subregion). The changing extent of the region that exceeds 10% K13 marker prevalence is summarised in (f). National shapefiles were obtained from the Malaria Atlas Project (MAP; https://malariaatlas.org/) under their open access policy (https://malariaatlas.org/open-access-policy/) and no changes were made.

Fig 3 shows that resistance is predicted to have spread and emerged geographically. This is further quantified in Fig 4, which highlights the geographical extent to which K13 prevalence is predicted to exceed 10% (based on the median estimates). The extent of resistance (using 10% K13 prevalence as a proxy) in 2000 is predicted to be isolated to near the Thai–Cambodia border and north Myanmar (Fig 4A). In the subsequent 5-year intervals, more of the region is predicted to exceed 10% K13 marker prevalence (see Fig 4B–4E showing the extent in 2005, 2010, 2015 and 2020, respectively). Fig 4F summarises the extent of resistance (at 10% K13 marker prevalence) over the Greater Mekong Subregion and shows that 2005 to 2015 demonstrate a substantial change in the extent. The extent of resistance at 50% K13 marker prevalence (panel (a) in Fig E in S1 Text) shows an isolated focal point near Pailin, Cambodia in 2003, then wide geographical change to 2010 and again to 2015. From 2015 to 2020 the extent of resistance (at 50% K13 marker prevalence) remains relatively static (see also panel (c) in Fig E in S1 Text, purple line). A second focal point in north Myanmar appears in 2009. The extent of resistance at 80% K13 marker prevalence (panel (b) in Fig E in S1 Text) isolates a focal point near Pailin, Cambodia, in 2010, some spatial change by 2015, after which it remains relatively fixed (see also panel (c) in Fig E in S1 Text, blue line). S4-5 and 6 Videos show, respectively, the changing extent of resistance at 10%, 50% and 80% K13 marker prevalence over 2000–2022. There is notable uncertainty in the proportion of the region that exceeds K13 marker prevalence of 10%, 50% and 80% over time (panel (c) in Fig E in S1 Text); however, the increasing trends are clear. Country level trends are broadly consistent with the trends for the whole region (Fig F in S1 Text). Cambodia is predicted to reach larger extents of resistance before the other countries in the region.

Using predicted median prevalence values for the region, we cluster points that exceed 10%, 50% and 80% K13 marker prevalence in space (S7-8 and 9 Videos respectively), visualising only those clusters that appear in more than a single year. At 10%, three existing clusters in 2000 located at the Thai–Cambodia border, north Myanmar and south Thailand grow and merge into one cluster encompassing most of the region by 2012. At 50%, a small cluster is established in 2004 near the Thai–Cambodia border, which expands over time, eventually encompassing several emerging clusters in the region. At 80%, a cluster emerges in 2010 and persists over time near the Thai–Cambodia border.

## Discussion

This paper has provided the first data-driven, predictive maps of the changing landscape of resistance to artemisinin derivatives in the Greater Mekong Subregion. These maps provide estimates of drug resistance for locations where no data are available and can be used by health agencies to guide the prioritisation of surveillance for resistance and policies to improve treatment and prevent the further spread of resistance.

The work presented in this paper significantly advances earlier work by providing predictions of K13 prevalence over the entire Greater Mekong Subregion from 2000 to 2022 based on a comprehensive dataset of K13 studies conducted in the region. It illustrates the expansion of artemisinin resistance in the Greater Mekong Subregion in the last 10 years and the increase of prevalence in east and north Myanmar, bordering Indian States. In 2015, Tun *et al*. quantified the spread of K13 propeller mutations with a similar Bayesian model–based geostatistics framework [15]; however, predictions were only for Myanmar in 2014 and based on the limited amount of available data at the time. The results presented in our paper are consistent with this earlier modelling work as well as the underlying data collected to assess the spread of artemisinin resistance (e.g. [4,5]).

The identification of mutations in the K13 protein that are associated with artemisinin resistance in 2014 provided a means for monitoring artemisinin resistance on a large geographical scale. Almost 200 different candidate mutant genotypes were first established [11,12]; meanwhile, a study from WWARN has since identified a refined set of K13 mutant alleles associated with slow parasite clearance–the clinical hallmark of artemisinin resistance [12]. This paper has presented maps for the prevalence of any of the K13 mutations associated with slow parasite clearance (as defined in [12]), thereby reducing the many markers to a single number of ‘positive’ observations for each study site. In this way, some of the statistical power that is available in the data is lost. The discovery of a set of molecular markers of resistance in the *K13* gene drives the need for innovation in the model structure that can capture the dependence between the markers. This will be possible with a joint statistical framework drawing on all the information available for each of the K13 markers for which the correlation structure between the markers is explicitly modelled; currently there is very limited data with more than one molecular marker, so a joint model is beyond the scope of this paper with the existing data. Furthermore, there is currently insufficient statistical power for a model for each marker.

We have characterised the changes in artemisinin resistance in terms of the prevalence of the K13 markers. Moreover, we have incorporated several key factors (transmission, human population, temperature suitability and accessibility) as covariates in the mean of the Gaussian process. However, these factors each have an important mechanistic role to play in the rate of change of artemisinin resistance, which could be modelled by considering another hierarchical layer in the statistical model that relates the level of resistance to, for example, the incidence of *Pf* malaria during a time interval.

There are several limitations to the work presented here. Firstly, the timeline from field data collection to publication to data sharing was often long; consequently, the modelling to provide real-time (or near real-time) information for policymakers was dependent on this factor and the willingness of all actors to allow rapid access to relevant data. It is worth noting that a pilot project conducted by Mahidol University and the WWARN from 2016 to 2019 had a turn-around time of three months for data upload onto publicly accessible maps [16]. This issue is highlighted by a known downward trend in K13 mutant infections in Cambodia since 2019 –likely driven by the change in first-line treatment–that is yet to be reflected in the data stored in WWARN repositories. Secondly, the modelling approach did not allow us to distinguish between multiple emergences and spread; the maps can represent only the extension of resistance through both combined. Thirdly, different studies have collected data on different sets of markers; a modelling approach would ideally consider the spatio-temporal changes in prevalence of each marker using a multi-output model. Fourthly, uncertainty exists that is overlooked that ideally should be incorporated into the model framework, in particular uncertainty concerning malaria transmission estimates. Additionally, other potential explanatory variables were not incorporated as covariates, because of a lack of sufficiently high-resolution spatial estimates. These include, but are not limited to, population movement, antimalarial treatment access and use (including asymptomatic carriers), and first-line antimalarial drug policy. Finally, the mediocre performance of the spatially explicit cross validation compared to the random approach is worth further exploration. With the sparsity of spatial data currently available this poor performance of long-range extrapolation is not unexpected [17] and may be improved as (1) more spatial data is collected, (2) modelling methods are improved for example to consider multi-model outputs, and (3) potential explanatory variables become available at sufficiently high spatiotemporal resolution.

This paper has confirmed the spatio-temporal patterns of drug resistance to artemisinin derivatives in Southeast Asia, where malaria elimination by 2030 is being actively targeted. The current elimination targets need to withstand the challenge of resistance to the current gold standard group of antimalarial drugs, artemisinin derivatives, that first emerged in Cambodia. Such resistance has now been detected in the Greater Mekong Subregion [4, 5]. Of extreme concern is that there are signs of emergence in Africa [18–20]. The consequences of artemisinin resistance may be dire: historically, the emergence and spread of parasites resistant to the antimalarial drugs chloroquine and later SP resulted in dramatic increases in malaria-related morbidity and mortality across sub-Saharan Africa [1]. If artemisinin resistance becomes established in Africa, it will likely facilitate the emergence and spread of ACT partner drug resistance, jeopardising the efficacy of ACTs, which will lead to an increase in malaria cases and, ultimately, an increase in malaria-attributable deaths.

The maps presented in this paper provide predicted artemisinin resistance levels in places and at times for which no data are available. The maps and model output can be used to help inform recommendations for appropriate drug choices at the local and regional levels. Furthermore, the success of existing resistance containment strategies can be assessed using the model by considering the predictions of the changing extent of resistance. The maps and model outputs are available to share with regional national malaria control programs to facilitate spatial decision support, including country and provincial level predictions. The approach presented here can naturally be extended to resistance of partner drugs (e.g. plasmepsin and PfCRT for piperaquine and PfMDR1 for mefloquine) to provide insights into ACT resistance [21], which would be most interesting for policymakers.

## Supporting information

S1 Text Contains supplementary methods and supplementary figures. *Fig A*. Covariates used in modelling. (a) Predicted parasite rate in 2–10 year olds in 2000, 2005, 2010 and 2015, (b) human population density, (c) *P*. *falciparum* temperature suitability, (d) travel time to nearest city as measure of accessibility. National shapefiles were obtained from the Malaria Atlas Project (MAP; https://malariaatlas.org/) under their open access policy (https://malariaatlas.org/open-access-policy/) and no changes were made. *Fig B*. Conditional dependency schematic for the geostatistical model. Here, solid arrows represent conditional dependencies, the dashed arrow represents a deterministic relationship, the squares represent data and the circles/ellipses represent random variables. *Fig C*. Posterior predictive median prevalence of K13 marker in the Greater Mekong Subregion in 2000 (a), 2010 (b) and 2020 (c). Associated standard deviations for posterior predictions in 2000 (d), 2010 (e) and 2020 (f). National shapefiles were obtained from the Malaria Atlas Project (MAP; https://malariaatlas.org/) under their open access policy (https://malariaatlas.org/open-access-policy/) and no changes were made. *Fig D*. The predicted area in the Greater Mekong subregion with K13 marker prevalence exceeding 10% (shaded region), based on median predictions, in 2000 (a), 2005 (b), 2010 (c), 2015 (d) and 2020 (e). The changing extent of the region that exceeds 10% K13 marker prevalence is summarised in (f). National shapefiles were obtained from the Malaria Atlas Project (MAP; https://malariaatlas.org/) under their open access policy (https://malariaatlas.org/open-access-policy/) and no changes were made. *Fig E*. The changing extent of the Greater Mekong subregion that exceeds 50% (a) and 80% (b) K13 marker prevalence. The proportion of the region with K13 marker prevalence exceeding 10%, 50% and 80% over the time period of 2000 to 2022 (c) where the median estimates are shown in the solid, coloured lines and the associated uncertainty (50% credible intervals) in the shaded regions. National shapefiles were obtained from the Malaria Atlas Project (MAP; https://malariaatlas.org/) under their open access policy (https://malariaatlas.org/open-access-policy/) and no changes were made. *Fig F*. The proportion of the Greater Mekong subregion with K13 marker prevalence exceeding 10%, 50% and 80% over the time period of 2000 to 2022 for Cambodia (a), Laos (b), Myanmar (c), Thailand (d), Vietnam (e) and the whole region (f). The median estimates are shown in the solid, coloured lines and the associated uncertainty (50% credible intervals) in the shaded regions. *Fig G*. Validation results showing (a) scatterplot of the predicted median prevalence from the validation models and observed prevalence in hold-out data and (b) probability-probability plot of the fraction of observations that fell within a predictive credible interval of a given size. The dashed red lines show a 1:1 reference line. In (a), the size of the dot is proportional to the sample size of the study. Table A. Summary of hyperparameters and prior choices in hierarchical model.
(DOCX)

S1 Video *K13* data collection over time. The video shows the time course of data collection for K13 over the period of 2000 to 2022. Data visualized in each year shows studies conducted before or during the year associated with the map. National shapefiles were obtained from the Malaria Atlas Project (MAP; https://malariaatlas.org/) under their open access policy (https://malariaatlas.org/open-access-policy/) and no changes were made.
(MP4)

S2 Video Spatiotemporal modelling of K13 mutation prevalence. The video shows the median of the posterior predictive distribution for K13 mutation prevalence over 2000 to 2022. National shapefiles were obtained from the Malaria Atlas Project (MAP; https://malariaatlas.org/) under their open access policy (https://malariaatlas.org/open-access-policy/) and no changes were made.
(MP4)

S3 Video Spatiotemporal modelling of K13 mutation uncertainty. The video shows the standard deviation of the posterior predictive distribution for K13 mutation prevalence over 2000 to 2022. National shapefiles were obtained from the Malaria Atlas Project (MAP; https://malariaatlas.org/) under their open access policy (https://malariaatlas.org/open-access-policy/) and no changes were made.
(MP4)

S4 Video Spatiotemporal modelling of K13 mutation prevalence at 10% threshold. The video shows the changing spatial extent of where resistance has reached 10% K13 marker prevalence over 2000 to 2022. National shapefiles were obtained from the Malaria Atlas Project (MAP; https://malariaatlas.org/) under their open access policy (https://malariaatlas.org/open-access-policy/) and no changes were made.
(MP4)

S5 Video Spatiotemporal modelling of K13 mutation prevalence at 50% threshold. The video shows the changing spatial extent of where resistance has reached 50% K13 marker prevalence over 2000 to 2022. National shapefiles were obtained from the Malaria Atlas Project (MAP; https://malariaatlas.org/) under their open access policy (https://malariaatlas.org/open-access-policy/) and no changes were made.
(MP4)

S6 Video Spatiotemporal modelling of K13 mutation prevalence at 80% threshold. The video shows the changing spatial extent of where resistance has reached 80% K13 marker prevalence over 2000 to 2022. National shapefiles were obtained from the Malaria Atlas Project (MAP; https://malariaatlas.org/) under their open access policy (https://malariaatlas.org/open-access-policy/) and no changes were made.
(MP4)

S7 Video Spatiotemporal clustering of K13 mutation prevalence at 10% threshold. The video shows the spatiotemporal clustered points that exceed 10% K13 marker prevalence in space over 2000 to 2022. National shapefiles were obtained from the Malaria Atlas Project (MAP; https://malariaatlas.org/) under their open access policy (https://malariaatlas.org/open-access-policy/) and no changes were made.
(MP4)

S8 Video Spatiotemporal clustering of K13 mutation prevalence at 50% threshold. The video shows the spatiotemporal clustered points that exceed 50% K13 marker prevalence in space over 2000 to 2022. National shapefiles were obtained from the Malaria Atlas Project (MAP; https://malariaatlas.org/) under their open access policy (https://malariaatlas.org/open-access-policy/) and no changes were made.
(MP4)

S9 Video Spatiotemporal clustering of K13 mutation prevalence at 80% threshold. The video shows the spatiotemporal clustered points that exceed 80% K13 marker prevalence in space over 2000 to 2022. National shapefiles were obtained from the Malaria Atlas Project (MAP; https://malariaatlas.org/) under their open access policy (https://malariaatlas.org/open-access-policy/) and no changes were made.
(MP4)
