# Supplementary material for: Spatio-temporal spread of artemisinin resistance in Southeast Asia
Source: PLoS Comput Biol. 2024 Apr 16;20(4):e1012017. doi: 10.1371/journal.pcbi.1012017 (PMC11051648; doi:10.1371/journal.pcbi.1012017)
Supplement: S1 Text — Fig A. Covariates used in modelling. (a) Predicted parasite rate in 2–10 year olds in 2000, 2005, 2010 and 2015, (b) human population density, (c) P. falciparum temperature suitability, (d) travel time to nearest city as measure of accessibility. National shapefiles were obtained from the Malaria Atlas Project (MAP; https://malariaatlas.org/) under their open access policy (https://malariaatlas.org/open-access-policy/) and no changes were made. Fig B. Conditional dependency schematic for the geostatistical model. Here, solid arrows represent conditional dependencies, the dashed arrow represents a deterministic relationship, the squares represent data and the circles/ellipses represent random variables. Fig C. Posterior predictive median prevalence of K13 marker in the Greater Mekong Subregion in 2000 (a), 2010 (b) and 2020 (c). Associated standard deviations for posterior predictions in 2000 (d), 2010 (e) and 2020 (f). National shapefiles were obtained from the Malaria Atlas Project (MAP; https://malariaatlas.org/) under their open access policy (https://malariaatlas.org/open-access-policy/) and no changes were made. Fig D. The predicted area in the Greater Mekong subregion with K13 marker prevalence exceeding 10% (shaded region), based on median predictions, in 2000 (a), 2005 (b), 2010 (c), 2015 (d) and 2020 (e). The changing extent of the region that exceeds 10% K13 marker prevalence is summarised in (f). National shapefiles were obtained from the Malaria Atlas Project (MAP; https://malariaatlas.org/) under their open access policy (https://malariaatlas.org/open-access-policy/) and no changes were made. Fig E. The changing extent of the Greater Mekong subregion that exceeds 50% (a) and 80% (b) K13 marker prevalence. The proportion of the region with K13 marker prevalence exceeding 10%, 50% and 80% over the time period of 2000 to 2022 (c) where the median estimates are shown in the solid, coloured lines and the associated uncertainty (50% credible intervals) in the [file pcbi.1012017.s001.docx]

**Supplementary Methods**

***Covariate data***

Our model makes use of 4 spatiotemporal covariates in the geostatistical model (see next subsection for details) which are summarised in Fig A. *P. falciparum* transmission intensity from 2010-2019 has been estimated by the spatiotemporal models developed by the Malaria Atlas Project (MAP) [1]. Fig A(a) shows *P. falciparum* parasite rate (PfPR; the proportion of the population found to carry asexual blood-stage parasites) in 2-10 years olds predicted by MAP for 2000, 2005, 2010 and 2015. Note that this may not be the most relevant parasite rate for the Greater Mekong Subregion where transmission is mostly in adults; however, it is likely to capture the spatial pattern of the parasite rate in adults and is the only publicly available measure of *P. falciparum* transmission available at an appropriate spatial and temporal scale.

Human population density estimates for the region of interest, taken from the WorldPop Project [2], are shown in Fig A(b) while temperature suitability for *P. falciparum* transmission is available from MAP (Fig A(c)) based on modelling from Gething *et. al* [3]. A global map of travel time (in minutes) to nearest city is a measure of accessibility is available from MAP (Fig A(d)) [4]. The covariates of parasite rate in the population group of 2-10 years old and temperature suitability are measured on a proportion scale ([0,1]) while population and accessibility are non-negative.

***Geostatistical Model***

Using a hierarchical model (Fig B), the number of individuals in the $i^{th}$study, conducted at location $x_{i}$ in year $t_{i}$, that were positive for the K13 marker ($N_{i}^{+}$) was taken to be binomially distributed, given the number of individuals tested in the $i^{th}$study ($N_{i}$) and the probability $p(x_{i},t_{i})$:

$N_{i}^{+} \sim\mathrm{Binomial}\left( N_{i}, p(x_{i},t_{i}) \right)$.

The probability, $p(x,t)$, at location$x$and time $t$, was modelled as the inverse logit transformation of a Gaussian Process (GP), $f(x,t)$, that allows spatial correlation to be captured:

$p\left( x,t \right)=\mathrm{logit}^{-1}\left( f\left( x,t \right) \right)$,

$f\left( x,t \right)|\theta_{M},\theta_{C}, \sim GP\left( \mu\left( x,t \right), C\left( x,t \right) \right)$,

where $\theta_{M}$ and $\theta_{C}$ are vectors of parameters specifying the mean, $\mu(x,t)$, and covariance function$, C(x,t)$, respectively. Predictive maps of $p(x_{i},t_{i})$ can then be made in the year/s and geographical location/s of interest.


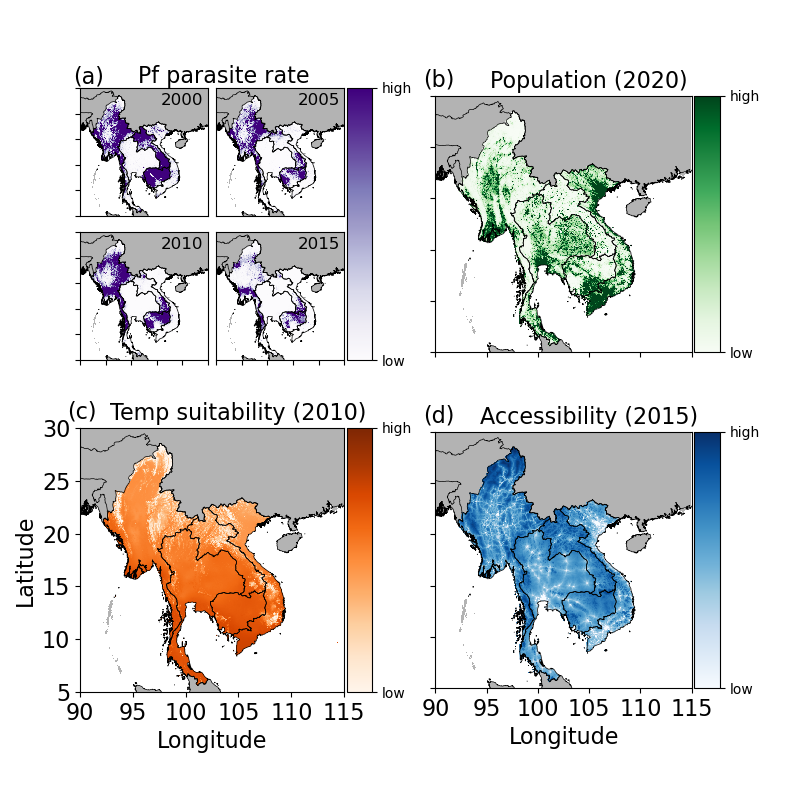


**Fig A**. Covariates used in modelling. (a) Predicted parasite rate in 2-10 year olds in 2000, 2005, 2010 and 2015, (b) human population density, (c) *P. falciparum* temperature suitability, (d) travel time to nearest city as measure of accessibility. National shapefiles were obtained from the Malaria Atlas Project (MAP; https://malariaatlas.org/) under their open access policy (https://malariaatlas.org/open-access-policy/) and no changes were made.


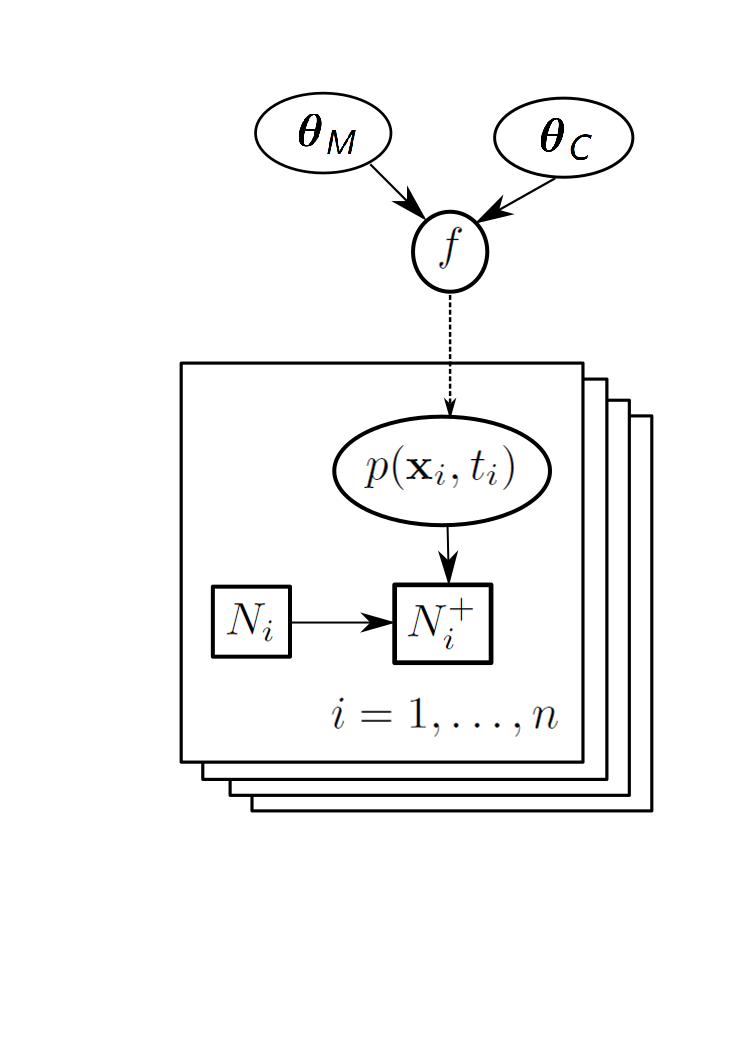


**Fig B**. Conditional dependency schematic for the geostatistical model. Here, solid arrows represent conditional dependencies, the dashed arrow represents a deterministic relationship, the squares represent data and the circles/ellipses represent random variables.

In this work, it was assumed that the mean function varied linearly with respect to time, $t$, malaria parasite rate, $M\left( x, t \right)$, temperature suitability, $T\left( x \right)$, transformed population density,$\mathrm{pop}\left( x \right)$, and transformed travel time accessibility, $A\left( x \right)$:

$$\mu\left( x,t \right)= \beta_{0}+ \beta_{1}t+ \beta_{2}M\left( x, t \right)+ \beta_{3}T\left( x \right)+ \beta_{4}\log\left( 1+pop\left( x \right) \right)+\beta_{5}\log\left( 1+A\left( x \right) \right).$$

We therefore have $\theta_{M}=\left\{ \beta_{0},\beta_{1},\beta_{2}, \beta_{3},\beta_{4},\beta_{5} \right\}$. Note that for years before 2010, we use the 2010 parasite rate and for years after 2020, we use the 2020 parasite rate.

The covariance function was chosen to be a version of the spatio-temporal structure advocated by Stein [5] and adopted previously [3, 6-8]. The covariance between two studies conducted at locations $x_{i}$ and $x_{j}$ and in years $t_{i}$ and $t_{j}$ was taken to be:

$C\left( x_{i},t_{i}, x_{j},t_{j} \right)= \sigma^{2}\gamma\left( 0 \right)\frac{{\Delta x}^{\gamma(\Delta t)}\kappa_{\gamma(\Delta t)}(\Delta x)}{2^{\gamma\left( \Delta t \right)-1}\Gamma(\gamma\left( \Delta t \right)+1)}+V\delta_{ij}$,

where $\Gamma$ is the gamma function,$\kappa_{\gamma}$ is the modified Bessel function of the second kind of order$\gamma$, $\Delta t=\left| t_{i}-t_{j} \right|$, $\gamma\left( \Delta t \right)=\left( 2\rho+2\left( 1-\rho\right)^{\frac{-\Delta t}{\phi_{t}}} \right)^{-1},$ $V$ is the nugget variance and $\delta_{ij}$ is a Kronecker delta that takes the value 1 if and only if $i=j$ and 0 otherwise. The distance$\Delta x$ is given by:

$\Delta x= \frac{2\sqrt{\gamma\left( \Delta t \right)}D_{GC}\left( x_{i}, x_{j} \right)}{\phi_{x}}$,

where $D_{GC}\left( x_{i}, x_{j} \right)$is the great circle distance between locations $x_{i}$ and $x_{j}$. In the notation adopted here, the parameter $\phi_{t}$refers to the temporal scale factor, $\rho$to the temporal limiting correlation, $\sigma^{2}$to the partial sill and $\phi_{x}$to the spatial range. The covariance parameters were thus ${\theta_{C}=\{\phi}_{t}, \phi_{x}, \rho, \sigma^{2}, V\}.$

The joint probability model for the $n$marker observations, the field components and the model parameters was therefore given by:

$p(N^{+}, f, \theta_{M}, \theta_{C})= \prod_{i=1}^{i=n} p\left( N_{i}^{+} | f\left( x_{i}, t_{i} \right), N_{i} \right)p\left( f | \theta_{M}, \theta_{C} \right)p\left( \theta_{M} \right)p\left( \theta_{C} \right)$.

Priors were specified for the mean and covariance parameters $\left\{ \theta_{M}, \theta_{C} \right\}=\{\beta_{0},\beta_{1},\beta_{2}, \beta_{3},\beta_{4},\beta_{5},\phi_{t}, \rho, \sigma^{2}, \phi_{x}, V\}$, see Table A. The logarithm of the partial sill ($\sigma^{2}$) and the spatial range ($\phi_{x}$) were assigned skew-normal priors, while the temporal scale ($\phi_{t}$) was given a relatively vague Exponential prior. The temporal limiting correlation ($\rho$) was assigned a uniform prior on $\left[ 0,1 \right]$ while improper flat priors were specified for the mean coefficients:

$p\left( \beta_{0},\beta_{1},\beta_{2}, \beta_{3},\beta_{4},\beta_{5} \right)\propto1$.

Finally, the inverse of the nugget variance ($V$) was assigned a diffuse Gamma prior.

| Parameter | Prior |
| --- | --- |
| Logarithm of square root of partial sill, $\sigma$ | SkewNormal (0.0535,1.79,3.21) |
| Logarithm of spatial range, $\phi_{x}$ (units of $\phi_{x}$are radians) | SkewNormal (-2.54, 1.42, -0.15) |
| Temporal scale, $\phi_{t}$ (units of $\phi_{t}$are years) | Exponential (0.1) |
| Temporal limiting correlation, $\rho$ | Uniform (0,1) |
| Inverse of nugget variance, $V^{-1}$ | Gamma (0.001, 0.004) |
| Mean (regression) parameters, $\beta_{0},\beta_{1},\beta_{2}, \beta_{3},\beta_{4},\beta_{5}$ | Improper flat priors |

**Table A**. Summary of hyperparameters and prior choices in hierarchical model.

The implementation of the model proceeds with two main steps: inference and prediction.

In the parameter estimation stage, the output of the model was the posterior probability distribution of the model parameters, given the observed data. Samples were drawn from the posterior distribution of the model mean and covariance parameters ($\theta_{M}, \theta_{C}$) and the random field ($f\left( x_{i}, t_{i} \right)$) at each location where the marker data was available, using an MCMC Metropolis-within-Gibbs approach. The MCMC algorithm was implemented in the Python package PyMC2; an open-source Python module [9] that implements Bayesian statistical models and fitting algorithms, including MCMC. The chain was run for 1,000,000 samples (burn-in set at 500,000). Convergence was visually assessed with autocorrelation and trace plots.

In the prediction stage, the output was the posterior predictive distribution of the prevalence of the marker at each space-time point of interest; here each location on a 5 x 5 km grid in the Greater Mekong Subregion from 2000-2022. From the output of the inference stage, parameter values were available for the sample from the posterior $\left\{ \beta_{0}^{j},\beta_{1}^{j}, \beta_{2}^{j},\beta_{3}^{j},\beta_{4}^{j},\beta_{5}^{j},\phi_{t}^{j}, \rho^{j},{\sigma^{2}}^{j}, \phi_{x}^{j}, V^{j} \right\}, j=1, \ldots, m$ and for $f^{j}\left( x_{i}, t_{i} \right), j=1, \ldots, m$ for each of the data locations ($i=1, \ldots, n$). To generate a predictive map for a year of interest, for each of the samples ($j=1, \ldots, m$), for each of the prediction locations on a 5 x 5 km grid, the conditional distribution of the random field, was sampled from a multivariable Normal distribution, conditional on $\left\{ \beta_{0}^{j},\beta_{1}^{j}, \beta_{2}^{j},\beta_{3}^{j},\beta_{4}^{j},\beta_{5}^{j},\phi_{t}^{j}, \rho^{j},{\sigma^{2}}^{j}, \phi_{x}^{j}, V^{j} \right\}$ and the $f^{j}\left( x_{i}, t_{i} \right), i=1, \ldots, n$. Repeating this for each of the $m$ samples formed the set of marker prevalence samples for this space-time location, for which the median and standard deviation was found. Repeating for each prediction location on a 5 x 5 km grid resulted in median and standard deviation maps of marker prevalence. For the predictive stage, $m=100$ samples were selected from the posterior samples (post burn-in).

***Clustering***

We use the clustering technique DBSCAN on the posterior median values to identify clusters of high prevalence [10]. We considered the posterior values greater than 10, 50 and 80 percentiles in the spatial domain to identify clusters. DBSCAN has two parameters: epsilon and minPts. Epsilon is a proximity parameter that defines the neighbourhood. A new point is added to a cluster if the distance from the new point to some other point in the cluster is less than epsilon. That is, if the new point is within an epsilon distance from the cluster. The parameter minPts defines the minimum number of points required to make a cluster. Points that do not belong to any cluster are considered noise. In our analysis, we have used epsilon = 50 km and minPts = 50, where each 5 x 5 km grid is considered a point.

***Model validation***

Model validity was assessed by dividing the dataset into 10 random subsets of data. For each subset, the model was rerun with the 10% of data withheld. The median predictions at the withheld points were then used as validation for the model. This was repeated for each of the 10 subsets of data. We report the Pearson correlation coefficient as a measure of linear association between the median predicted values and the observed prevalence, the mean error as a measure of bias and the mean absolute error as a measure of average accuracy.

In addition, we performed a spatially explicit cross validation by repeating the above process but with the data spatially clustered into 50 clusters. We used a kmeans clustering algorithm to split the data points into 50 clusters. The longitudes and latitudes of the data points were used for clustering. Thus, the clusters are location-based, not time-based. Due to the non-uniform spatial spread of the data points, certain clusters have many data points, while others have less (minimum cluster size was 1 and maximum size was 29).

These geostatistical models cannot extrapolate the spatial field very far reliably and so cannot be expected to accurately predict data values a long distance away from data points. When presenting the maps, we still include predictions to regions far from datapoints, since predictions in these areas are based on the environmental covariates. Whilst much less accurate, these are unfortunately the best predictions possible given the sparsity of the data. The significant uncertainty associated with these predictions is clearly displayed in the uncertainty maps that accompany these predictions. We therefore set up the spatial cross-validation scheme to evaluate the ability of the geostatistical model to perform its task of interpolating resistance over a moderate spatial range between datapoints in relatively well-sampled regions.

**Supplementary Figures**


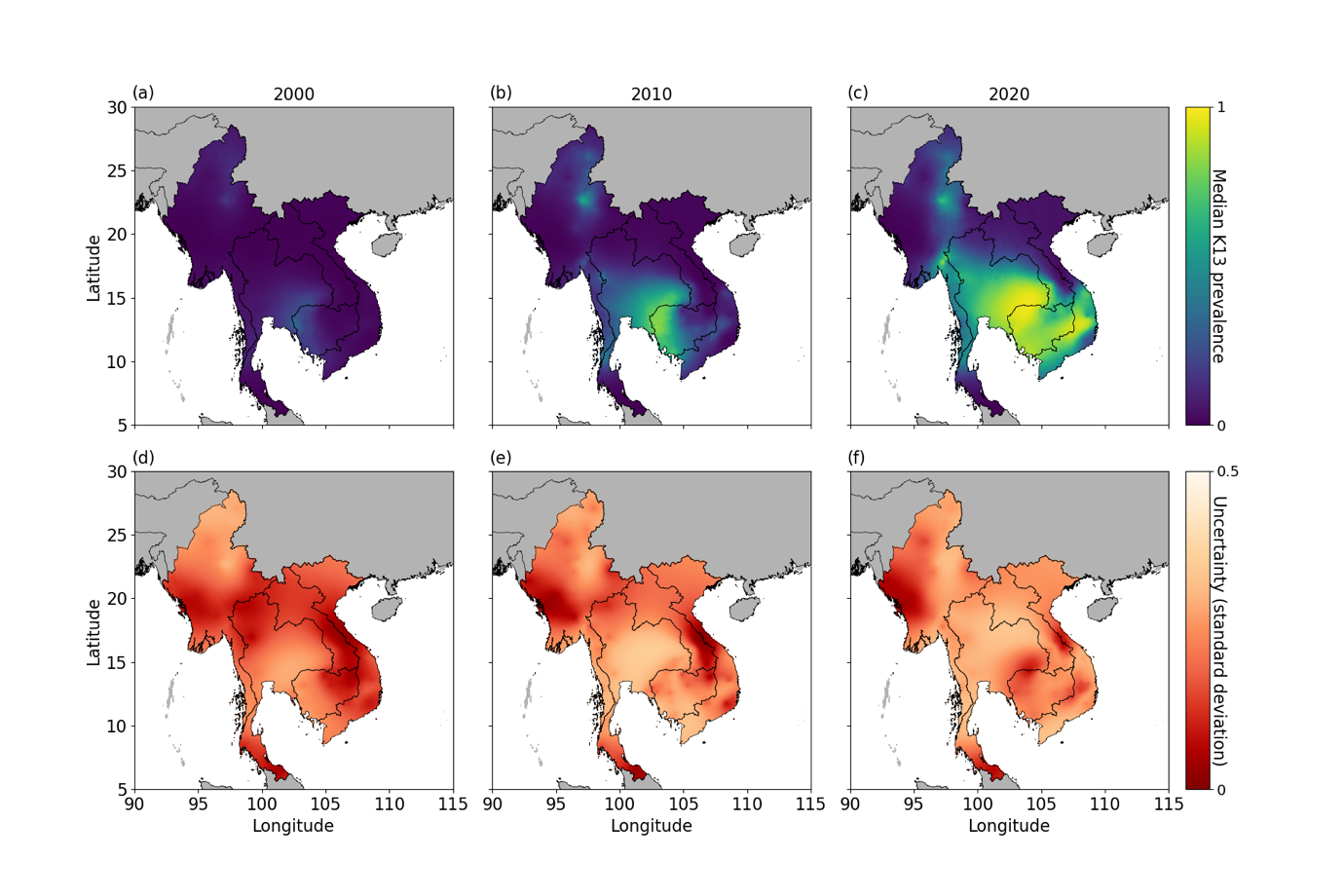


**Fig C**. Posterior predictive median prevalence of K13 marker in the Greater Mekong Subregion in 2000 (a), 2010 (b) and 2020 (c). Associated standard deviations for posterior predictions in 2000 (d), 2010 (e) and 2020 (f). National shapefiles were obtained from the Malaria Atlas Project (MAP; https://malariaatlas.org/) under their open access policy (https://malariaatlas.org/open-access-policy/) and no changes were made.


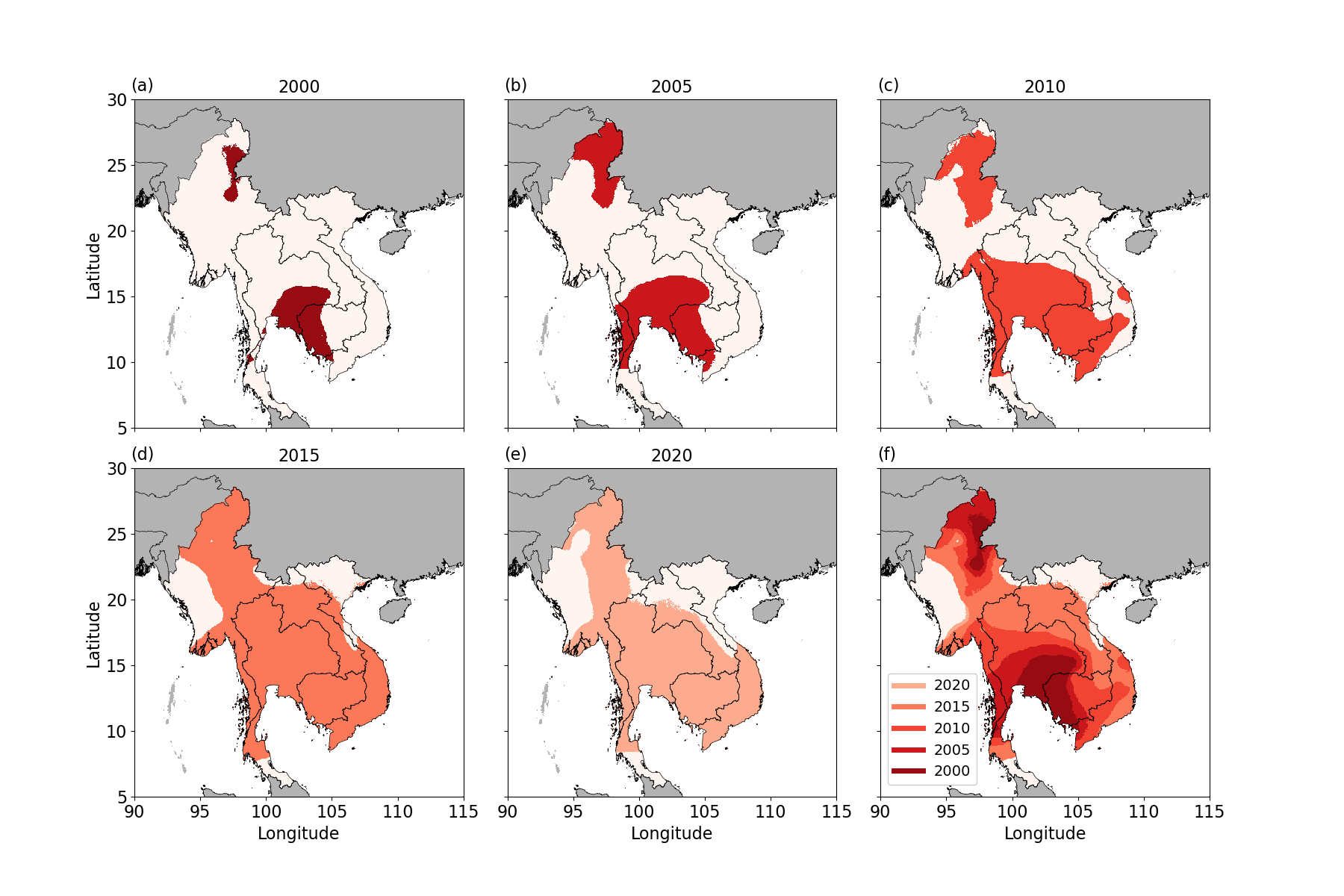


**Fig D**. The predicted area in the Greater Mekong subregion with K13 marker prevalence exceeding 10% (shaded region), based on median predictions, in 2000 (a), 2005 (b), 2010 (c), 2015 (d) and 2020 (e). The changing extent of the region that exceeds 10% K13 marker prevalence is summarised in (f). National shapefiles were obtained from the Malaria Atlas Project (MAP; https://malariaatlas.org/) under their open access policy (https://malariaatlas.org/open-access-policy/) and no changes were made.


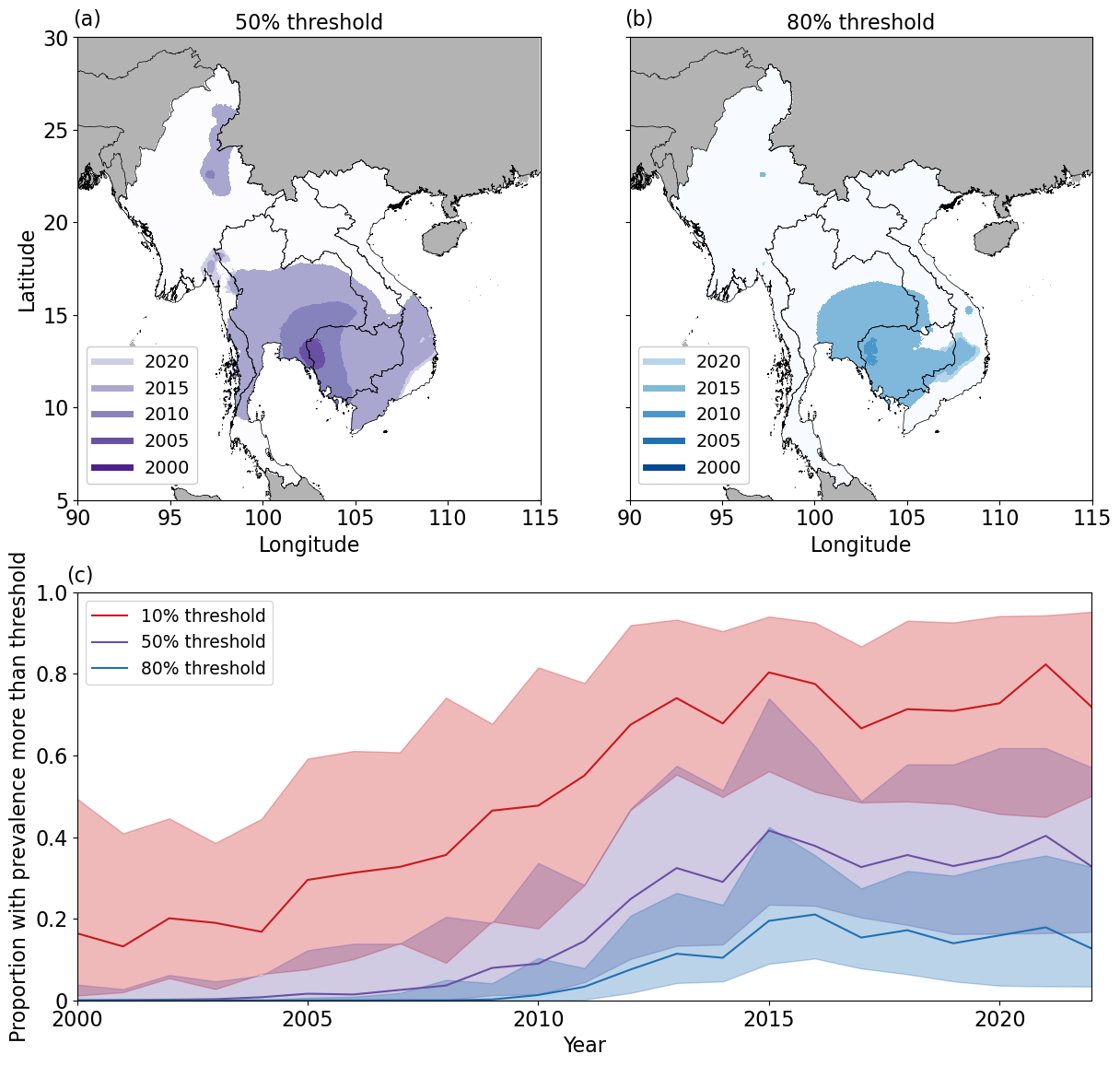
 **Fig E**. The changing extent of the Greater Mekong subregion that exceeds 50% (a) and 80% (b) K13 marker prevalence. The proportion of the region with K13 marker prevalence exceeding 10%, 50% and 80% over the time period of 2000 to 2022 (c) where the median estimates are shown in the solid, coloured lines and the associated uncertainty (50% credible intervals) in the shaded regions. National shapefiles were obtained from the Malaria Atlas Project (MAP; https://malariaatlas.org/) under their open access policy (https://malariaatlas.org/open-access-policy/) and no changes were made.


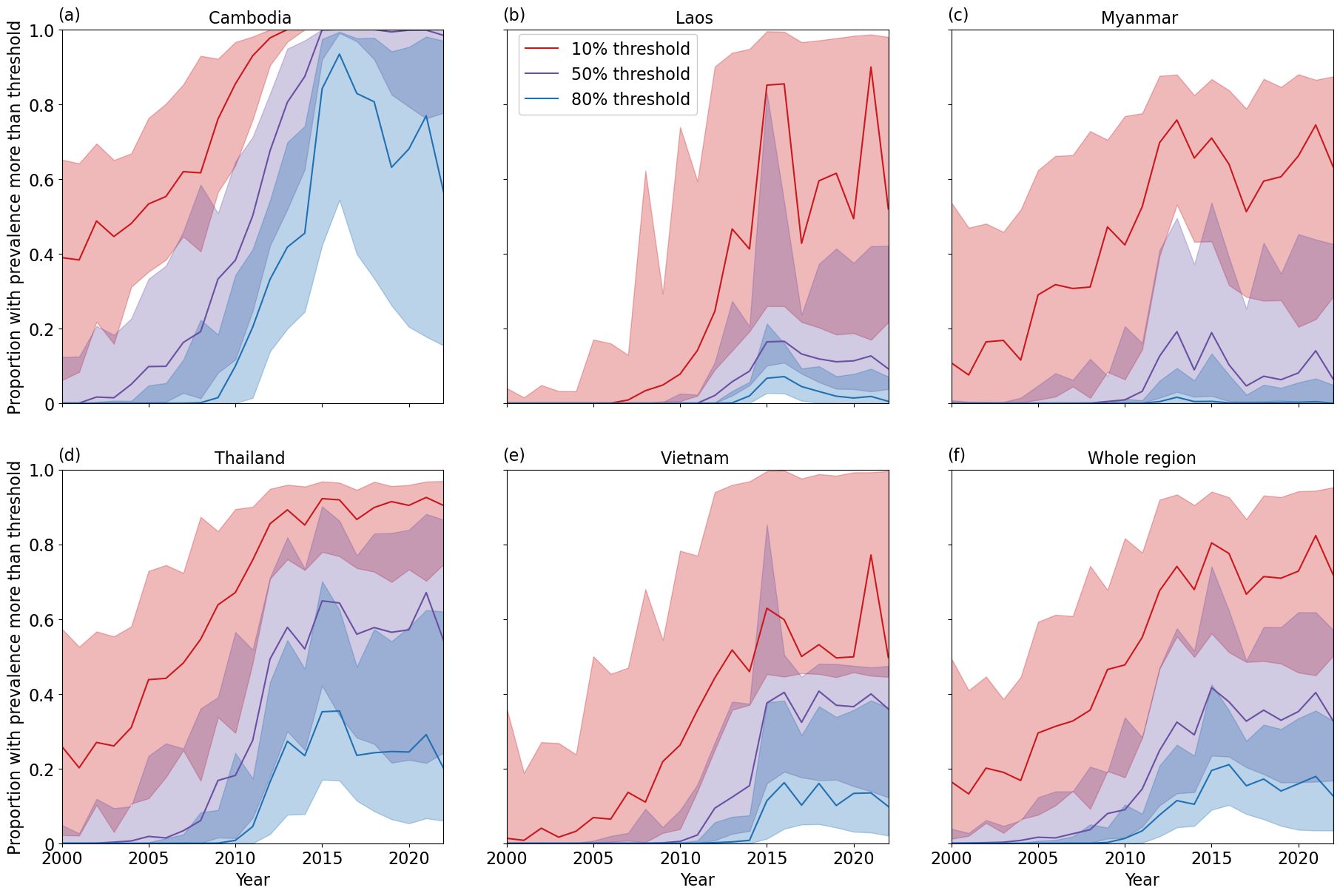


**Fig F**. The proportion of the Greater Mekong subregion with K13 marker prevalence exceeding 10%, 50% and 80% over the time period of 2000 to 2022 for Cambodia (a), Laos (b), Myanmar (c), Thailand (d), Vietnam (e) and the whole region (f). The median estimates are shown in the solid, coloured lines and the associated uncertainty (50% credible intervals) in the shaded regions.


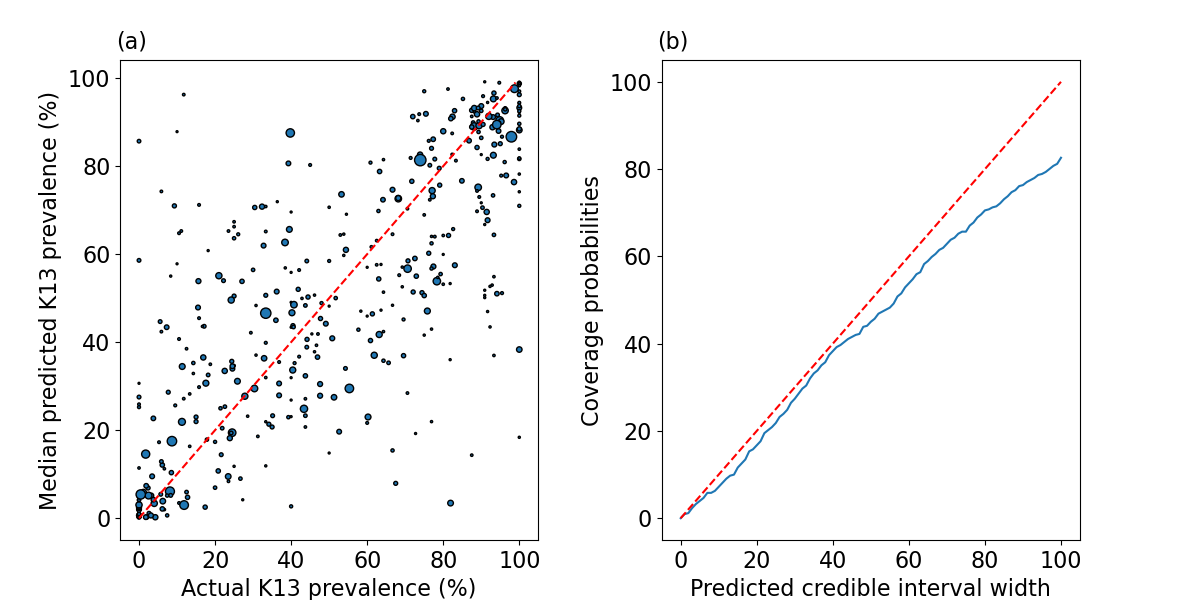


**Fig G**. Validation results showing (a) scatterplot of the predicted median prevalence from the validation models and observed prevalence in hold-out data and (b) probability-probability plot of the fraction of observations that fell within a predictive credible interval of a given size. The dashed red lines show a 1:1 reference line. In (a), the size of the dot is proportional to the sample size of the study.

**References**

1. *Malaria Atlas Project*. [cited 2020 May 1]; Available from: <https://malariaatlas.org/>.

2. *WordPop*. [cited 2020 May 1]; Available from: <https://www.worldpop.org/>.

3. Gething, P.W., T.P. Van Boeckel, D.L. Smith, et al., *Modelling the global constraints of temperature on transmission of Plasmodium falciparum and P. vivax.* Parasites & vectors, 2011. **4**(1): p. 1-11.

4. Weiss, D.J., A. Nelson, H. Gibson, et al., *A global map of travel time to cities to assess inequalities in accessibility in 2015.* Nature, 2018. **553**(7688): p. 333-336.

5. Stein, M.L., *Space–time covariance functions.* Journal of the American Statistical Association, 2005. **100**(469): p. 310-321.

6. Flegg, J.A., A.P. Patil, M. Venkatesan, et al., *Spatiotemporal mathematical modelling of mutations of the dhps gene in African Plasmodium falciparum.* Malaria Journal, 2013. **12**(1): p. 249.

7. Tun, K.M., M. Imwong, K.M. Lwin, et al., *Spread of artemisinin-resistant Plasmodium falciparum in Myanmar: a cross-sectional survey of the K13 molecular marker.* The Lancet Infectious Diseases, 2015. **15**(4): p. 415-421.

8. Hay, S.I., C.A. Guerra, P.W. Gething, et al., *A world malaria map: Plasmodium falciparum endemicity in 2007.* PLoS Med, 2009. **6**(3): p. e1000048.

9. Patil, A., D. Huard, and C.J. Fonnesbeck, *PyMC: Bayesian stochastic modelling in Python.* Journal of statistical software, 2010. **35**(4): p. 1.

10. Ester, M., H.-P. Kriegel, J. Sander, et al. *A density-based algorithm for discovering clusters in large spatial databases with noise*. in *Kdd*. 1996.
